# Interferon Regulatory Factors (*IRF1*, *IRF4*, *IRF5*, *IRF7* and *IRF9*) in Sichuan taimen (*Hucho bleekeri*): Identification and Functional Characterization

**DOI:** 10.3390/genes15111418

**Published:** 2024-10-31

**Authors:** Yeyu Chen, Huanchao Yang, Xiaoyun Wu, Zhao Liu, Yanling Chen, Qinyao Wei, Jue Lin, Yi Yu, Quanyu Tu, Hua Li

**Affiliations:** 1 Fisheries Research Institute, Sichuan Academy of Agricultural Sciences, Chengdu 611730, China; cyyleaf@scsaas.cn (Y.C.); yhc123@scsaas.cn (H.Y.); wuxy@scsaas.cn (X.W.); lz754299716@163.com (Z.L.); cqpcy123@scsaas.cn (Y.C.); weiqinyao@scsaas.cn (Q.W.); linjue@scsaas.cn (J.L.); 17784411664@163.com (Y.Y.); tuquanyu-12@163.com (Q.T.); 2 Fish Resources and Environment, The Upper Reaches of the Yangtze River Observation and Research Station of Sichuan Province, Chengdu 611730, China; 3 Key Laboratory of Bio-Resources and Eco-Environment of Ministry of Education, College of Life Sciences, Sichuan University, Chengdu 610065, China

**Keywords:** interferon regulatory factor, *Hucho bleekeri*, tissue distribution, immune response

## Abstract

**Background/Objectives**: Interferon regulatory factors (IRFs) are multifunctional transcription factors that play important roles in the transcriptional regulation of interferons and in the immune response to pathogens. Therefore, studying the interferon system in fish is highly relevant in the prevention and treatment of viral diseases. **Methods**: In this study, five IRF genes (*IRF1*, *IRF4*, *IRF5*, *IRF7* and *IRF9*) were identified and characterized in *Hucho bleekeri*, and their expression profiles were determined after LPS and Poly(I:C) treatment. **Results**: These IRFs have typical DNA-binding domains and IRF-association domains. Amino acid sequence comparison revealed high homology between these IRFs and those of other vertebrates, with the highest homology being with other salmonid fish. Phylogenetic analysis revealed that these IRFs are divided into four subfamilies (IRF1, IRF3, IRF4 and IRF5), with both IRF4 and IRF9 belonging to the IRF4 subfamily. IRF genes were widely expressed in all of the tested tissues, with *IRF1*, *IRF4* and *IRF9* being highly expressed in the spleen and kidney and *IRF5* and *IRF7* highly expressed in the gonads. *IRF1*, *IRF4* and *IRF5* expression was induced at different time points post-LPS challenge. *IRF7* and *IRF9* expression in the spleen and head kidney was not significantly altered by LPS induction. Poly(I:C) treatment altered *IRF* expression more significantly than LPS treatment. Poly(I:C) significantly altered the spleen and head kidney expression of all five *IRFs*. **Conclusions**: These findings reveal the potential role of IRFs in the antiviral response of *H. bleekeri* and provide a reference for examining signal transduction pathways in the interferon system in fish.

## 1. Introduction

Compared with the specific immune system, the innate immune system plays a greater role in resisting pathogen infection, especially against bacterial and viral diseases, which cause the greatest losses in aquaculture. When an external pathogen invades, the host recognizes the viral components via pattern recognition receptors, thereby activating the antiviral functions of the innate immune system. At least three families of pattern recognition receptors are known, including the retinoic acid-inducible gene I (RIG-I)-like receptor (RLR), Toll-like receptor (TLR) and nucleotide-binding and oligomerization domain-like receptor (NLR) families [1,2,3]. After recognizing the viral RNA, RLR is coupled to the IFN-β promoter stimulator-1 via the CARDs domain of the intracellular segment [4,5,6,7]. The interferon regulatory factor (IRF) gene is then phosphorylated via a series of signaling cascades, inducing interferon (IFN) production. The IFN system, an important part of the innate immune system, is the earliest cell-function regulatory system in the defense response [8] and is also the main mechanism of vertebrate resistance to viral infection. Timely induction of IFN expression is the primary condition for its antiviral effect. 

IRFs are transcription factors with important roles in regulating IFN. They are widely present in various cells and are central in immune responses, such as in resistance to pathogen infection, cell proliferation and apoptosis [9,10]. IRF recognizes the IFN-stimulated response elements A/GNGAAANNGAAACT in the promoter regions of IFNs and IFN-stimulated genes (ISGs), enabling them to regulate the expression of target genes [11]. The IRF genes of fish and mammals exhibit some homology, although their immune response functions differ substantially [12,13]. Fish and mammals have 9 and 11 IRF genes, respectively. The differences between fish and the higher vertebrates in immune recognition and activation processes remain unclear. 

Bioinformatics analysis has revealed that the IRF family in fish can be divided into four subfamilies: IRF1 (including IRF1, IRF2 and IRF11), IRF3 (including IRF3 and IRF7), IRF4 (including IRF4, IRF8, IRF9 and IRF10) and IRF5 (including IRF5 and IRF6) [14,15]. IRFs have been identified in several fish species, with their expression recorded in all organs or tissues examined in large yellow croaker (*Larimichthys crocea*) [16], red sea bream (*Pagrus major*) [17], common carp (*Cyprinus carpio* L.) [18,19], Asian seabass (*Lates calcarifer*) [20], miiuy croaker (*Miichthys miiuy*) [21], Pacific cod (*Gadus macrocephalus*) [22], half-smooth tongue sole (*Cynoglossus semilaevis*) [23], Atlantic cod (*Gadus morhua*) [24], sea perch (*Lateolabrax japonicus*) [25], mandarin fish (*Siniperca chuatsi*) [14] and golden pompano (*Trachinotus ovatus*) [26]. IRF gene expression in fish is significantly induced by viruses [25,27], bacteria [28,29], poly(I:C) [30,31], LPS treatment [28] and parasites [32], and their expression in immune tissues increases to different extents depending on the stage of infection.

Sichuan taimen (*H. bleekeri*) is a unique cold water fish species found in China. It is the southernmost of the five *Hucho* species, and its distribution expanded from the north as the climate cooled during the Quaternary glacial period. After the end of the glacial period, it remained in rivers with higher elevation and colder water [33,34,35], and is regarded as a typical landlocked fish. *H. bleekeri* is distributed in Sichuan (Dadu River, upper Minjiang River and its tributaries), Qinghai (Make River) and Shaanxi (Taibai and Xushui Rivers, tributaries of the upper Hanjiang River). However, since the 1960s, its population has declined dramatically owing to overfishing, environmental pollution and intensive development of water facilities [36], and it is currently difficult to find samples for collection in multiple parts of its historical distribution. It is listed as a national Class I protected animal in the newly released List of National Key Protected Wild Animals and as a critically endangered species in the IUCN Red List Book. This fierce apex predator, a flagship species in the upper reaches of the Yangtze River, plays an extremely important role in maintaining the ecological balance of the waters. However, it is at risk of extinction and can only be maintained via captive breeding. Disease prevention and control are important components of healthy breeding; nonetheless, few studies have addressed disease and immunity in this species.

Although IRFs participate in regulating type I IFNs, no studies have examined the immune function of IRF genes in *H. bleekeri*. To address this, we selected *IRF1*, *IRF4*, *IRF5*, *IRF7* and *IRF9* from four IRF subfamilies and analyzed their molecular characteristics and functions in the innate immune response. These results are important for disease prevention and control in *H. bleekeri* and will enhance our understanding of innate immune regulation in salmonid fish.

## 2. Materials and Methods

### 2.1. Animals

Sichuan taimen used in this study were obtained from the Jiguanshan Cold Water Fish Breeding Base of Sichuan Fishery Institute (Chengdu, China). After transport, they were temporarily kept in laboratory with dissolved oxygen greater than 5 mg/L at 12.8 ± 0.2 °C. The fish were observed for two weeks to ensure that their growth was normal. The fish were further screened to ensure they were free of *Aeromonas salmonicida* and *Yersinia ruckeri*.

All fish handling and experimental procedures were approved by the Animal Care and Use Committee of the Fishery Institute of the Sichuan Academy of Agricultural Sciences (20230308001A). All animal collection and use protocols were carried out in accordance with this institute’s guidelines and regulations for the care and use of laboratory animals.

### 2.2. Gene Identification and Biological Information Analysis

IRF cDNA sequences were obtained from our previous full-length transcriptome sequencing database of *H. bleekeri* [37]. To verify the sequence obtained from transcriptome sequencing, primers were designed to amplify the full-length open reading frame (ORF) sequences of *IRF1*, *IRF4*, *IRF5*, *IRF7* and *IRF9.* As the PCR template, we used spleen-derived cDNA of *H. bleekeri*. Total RNA of spleen was isolated using the TRIzol reagent (Invitrogen, Carlsbad, USA), following the manufacturer’s protocol. The quality of the extracted RNA was determined by gel electrophoresis and by detecting absorbance at A260/230 and A260/280. Total RNA was reverse-transcribed into the first strand of cDNA using the ReverTra Ace-First Strand cDNA Synthesis Kit (Takara, Otsu, Japan). PCR amplification products were detected by gel electrophoresis. The sizes of PCR products are listed in Table 1. The amplified products were connected to a pESI-T vector (30 ng/μL) and reacted at room temperature for 5 min. Then, competent cells were added for transformation. The positive clones were selected for sequencing with primers (M13F: TGTAAAACGACGGCCAGT; M13R: CAGGAAACAGCTATGACC).

The NCBI database was used to analyze IRF homology between *H. bleekeri* and other species. We utilized SMART v8 “http://smart.embl-heidelberg.de/ (accessed on 27 September 2024)”, along with multiple alignment using ClustalW v1.83, including sequences from other species, to predict the functional structure of IRF. Vector NTI software v11 was used to predict the open reading frame (ORF) region, molecular weight and isoelectric point. Phylogenetic analysis of IRF was performed using the neighbor-joining (NJ) method in MEGA 6.0. The tree was conducted using the bootstrap method with 1000 bootstrap replications. The Poisson model was selected in substitution analysis. The three-dimensional (3D) structures of the IRF proteins were constructed using Swiss-model (latest version) “https://swissmodel.expasy.org/ (accessed on 27 September 2024)”.

### 2.3. Tissue Expression Analysis

Three healthy *H. bleekeri* were selected and dissected on a sterilized dissection table, following anesthesia using MS-222 (Sigma Aldrich Co., St. Louis, MO, USA). Samples of skin, brain, gills, head kidney, trunk kidney, liver, spleen, eye, gonad, heart, stomach, duodenum, hindgut, rectum and muscle were selected. The samples were quickly packed in liquid nitrogen in 1.5 mL RNAse-free Eppendorf tubes and were stored at −80 °C.

Total RNA of 15 tissues were isolated as described in Section 2.2. The resulting cDNA was used for tissue expression analysis.

### 2.4. In Vitro Stimulation

Primary cells were utilized to examine IRF expression profiles following in vitro stimulation, as previously described [38]. The head kidneys and spleens of three healthy *H. bleekeri* were isolated on a clean bench. The tissues were cut into pieces in RPMI 1640 medium and filtered through a 100 μm nylon mesh cell strainer. The cells were centrifuged at 400× *g* for 10 min at 4 °C and the supernatant discarded. The cells were resuspended in RPMI 1640 medium containing 15% fetal calf serum. Trypan blue staining was used to assess cell viability. The cells were evenly distributed into six-well cell-culture plates and the medium was added to a final volume of 2 mL per well and final cell concentration of ca. 5 × 10^6^ cells/mL. The primary cells were stimulated with LPS (50 μg/mL, sourced from *Escherichia coli* 055:B5), poly(I:C) (100 μg/mL), or an equal volume of PBS as control, in triplicate. Cells were incubated at 13 °C with 5% CO_2_. The cells were harvested at 12, 24 and 48 h post-challenge. 

### 2.5. Real-Time PCR

Real-time PCR was performed using the Bio-Rad CFX Connect System (Bio-Rad, Hercules, CA, USA). The cDNA for real-time PCR analysis was diluted 20-fold. The cycling parameters were as follows: 3 min at 98 °C, followed by 40 cycles consisting of 5 s at 98 °C, 10 s at each gene’s annealing temperature (Table 1), and 15 s at 72 °C. Melting curve analysis was conducted over the temperature range 70–95 °C. *EF-1α* was used as the reference gene. Expression was analyzed using the 2^−ΔΔCT^ method. In the expression analysis of normal fish, the relative expression of IRFs in different tissues was normalized to that in tissues with the lowest expression; in the stimulation experiment, the relative expression of IRFs was normalized to the data for the control group.

### 2.6. Data Statistics

The expression levels of IRFs in different tissues of normal fish were analyzed by one-way analysis of variance (ANOVA) in SPSS 19.0. Differential expression of IRFs in the stimulation experiment was evaluated using an independent *t*-test in SPSS 19.0. Differences were considered significant at *p* < 0.05.

## 3. Results

### 3.1. IRF cDNA Sequence Characteristics

Based on full-length transcriptome sequencing [37], the IRF1 cDNA sequence is 1605 bp in length, with a 149 bp 5′ untranslated region (5′ UTR), a 939 bp ORF and a 517 bp 3′ untranslated region (3′ UTR). The 3′ UTR contains a polyadenylation signal (AATAAA). The verified ORF-encoded peptide comprises 312 amino acid residues with a presumed molecular weight of 35.6 kDa and an isoelectric point of 5.13. The cDNA length obtained by transcriptome sequencing of IRF4, IRF5, IRF7 and IRF9 are 2156 (50 bp 5′ UTR and 699 bp 3′ UTR), 2494 (388 bp 5′ UTR and 525 bp 3′ UTR), 1866 (76 bp 5′ UTR and 608 bp 3′ UTR) and 1528 bp (77 bp 5′ UTR and 128 bp 3′ UTR), respectively. The ORF lengths of IRF4, IRF5, IRF7 and IRF9 are 1407, 1581, 1182 and 1323 bp, encoding 468, 526, 393 and 440 amino acids, respectively. The presumed molecular weights of IRF4, IRF5, IRF7 and IRF9 are 53.4, 59.5, 45.4 and 49.3 kDa, respectively. The presumed isoelectric points of IRF4, IRF5, IRF7 and IRF are 5.7, 5.34, 5.98 and 6.33, respectively (Table S1).

### 3.2. Amino Acid Structures of IRF1, IRF4, IRF5, IRF7 and IRF9

**Figure 1:**
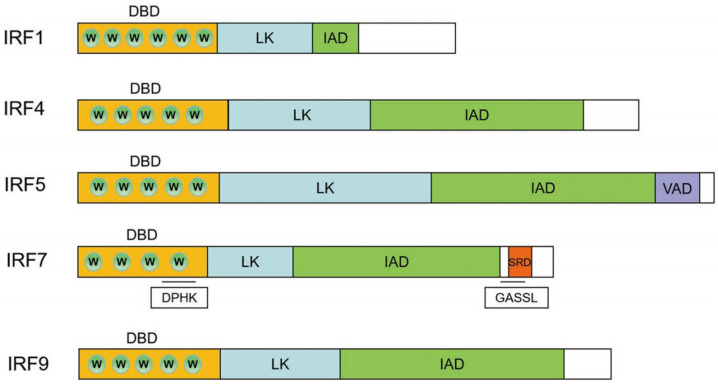
The features of amino acids structures in IRF1, IRF4, IRF5, IRF7 and IRF9. DBD: DBD-binding domain, W: tryptophan, LK: linker region, IAD: IRF association domain, VAD: virus-activated domain, SRD: serine-rich domain.

IRF1 in *H. bleekeri* is similar to that of other species. At its N-terminus, it contains a highly conserved DBD-binding domain (DBD) comprising 115 amino acid residues and six conserved tryptophan repeats (Trp11, Trp26, Trp38, Trp46, Trp58 and Trp77) (Figure 1). Following the DBD was a linker region (LK). In IRF1, the IRF association domain (IAD) is located in the middle of the peptide (at amino acids 195–232). In IRF4, the DBD (located in the N terminus) comprises the first 124 amino acids, with five conserved tryptophan repeats (Trp22, Trp37, Trp49, Trp69 and Trp88) (Figure 1); following the DBD was an LK, and the IAD is located at amino acids 247–422. The IRF5 DBD comprises 117 amino acids and five conserved tryptophan repeats (Trp14, Trp29, Trp41, Trp61 and Trp80) (Figure 1); its IAD contains 185 amino acids and is followed immediately by a virus-activated domain (VAD) of 37 amino acids. In IRF7, the DBD comprises 107 amino acids and four conserved tryptophan repeats (Trp11, Trp36, Trp55 and Trp72) (Figure 1); its IAD contains 171 amino acids and is followed by a serine-rich domain (SRD) of 19 amino acids. This SRD is located at the peptide’s C-terminus, and is similar in structure to the mammalian SRD. The IRF7 DBD contains a conserved DPHK domain, and a GASSL domain is located between the IAD and SRD. In IRF9, the DBD comprises 117 amino acids and five conserved tryptophan repeats (Trp15, Trp30, Trp42, Trp62 and Trp80) (Figure 1), and its IAD contains 185 amino acids.

**Figure 2:**
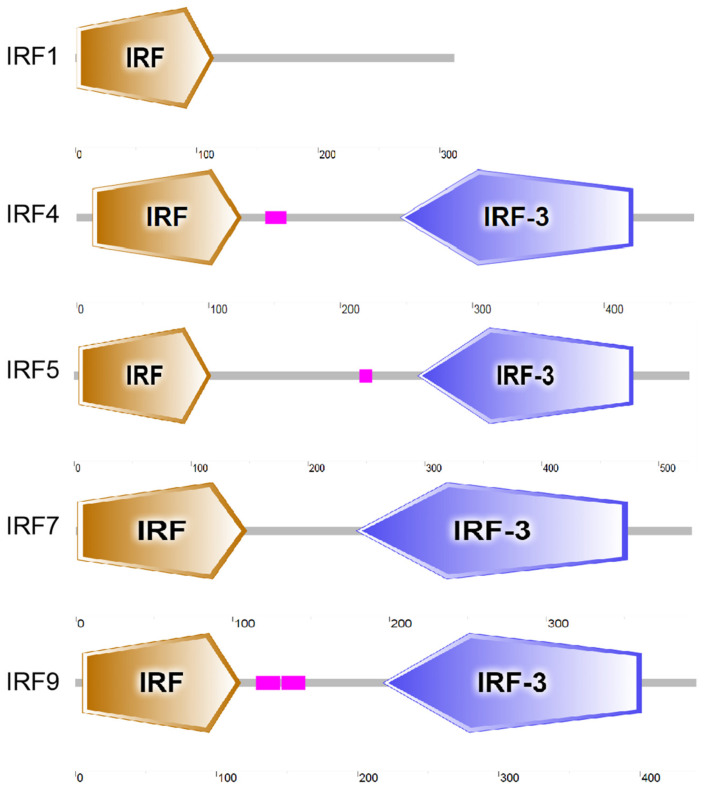
Schematic representation of IRF1, IRF4, IRF5, IRF7 and IRF9 predicted by SMART programs. Orange boxes indicated IRF domains, purple boxes in IRF4, IRF5 and IRF9 indicate low-complexity regions.

SMART-based prediction reveals that all five IRFs exhibit IRF structures, with E-values of 4.79 × 10^−44^, 3.82 × 10^−61^, 1 × 10^−55^, 4.73 × 10^−25^ and 1.84 × 10^−54^ for IRF1, IRF4, IRF5, IRF7 and IRF9, respectively. IRF4, IRF5, IRF7 and IRF9 exhibit IRF-3 domains, with E-values of 2 × 10^−61^, 2.2 × 10^−80^, 3.51 × 10^−65^ and 1.54 × 10^−43^, respectively (Figure 2).

### 3.3. 3D Protein Structure Prediction of IRF1, IRF4, IRF5, IRF7 and IRF9

**Figure 3:**
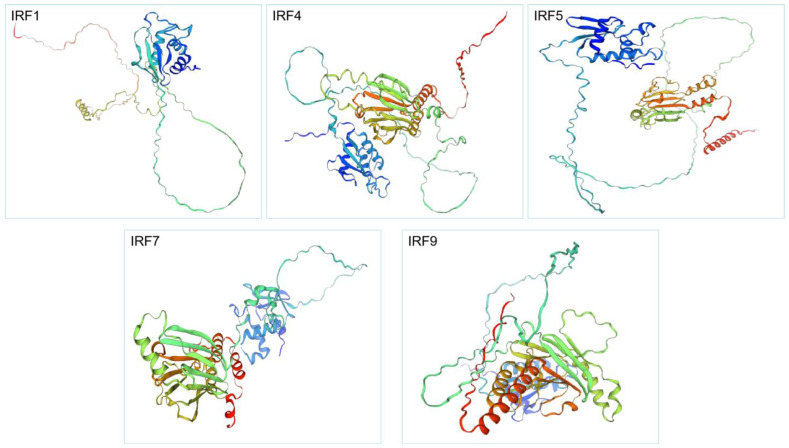
The three-dimensional structure of IRF proteins predicted by Swiss-model.

Based on 3D protein structure prediction, IRFs contain α-helix, strand and coil structures (Figure 3). In IRF1, the α3 helix domain contains four conserved amino acid residues (Arg82, Cys83, Asn86 and Ser87) that mediate IRFs binding to DNA. In IRF4, IRF5 and IRF9, lysine (Lys) has replaced Ser87, resulting in the arrangement Arg, Cys, Asn and Lys. In IRF7, Serine (S) has replaced Cys83, resulting in the arrangement of Arg, Ser, Asn and Ser.

### 3.4. IRF Amino Acid Homology Among Species

The level of identity of the IRF1 amino acid sequences between *H. bleekeri* and other fish species ranged from 67.8 to 87.5% (Table S2, Figure S1), with *Oncorhynchus keta* exhibiting the highest identity with *H. bleekeri*. *H. bleekeri* IRF1 and *Salmo* and *Oncorhynchus* species exhibited high IRF1 identity. *H. bleekeri* and mammalian IRF1 exhibited low identity, at 34.3% (with human IRF1) and 34.6% (with mouse IRF1).

IRF4 identity in *H. bleekeri* and other fish species ranged from 75.91 to 97.86%, with *Salvelinus namaycush* showing the highest identity. For human and mouse IRF4, the identity levels were 48.8 and 52.1%, respectively.

The IRF5 identity between *H. bleekeri* and other fish species, in terms of the amino acid sequence, ranged from 67.98 to 94.74%, with *Salmo salar* exhibiting the highest identity with *H. bleekeri*. The identities of *H. bleekeri* IRF5 with human and mouse IRF5 were 46.6 and 47.1%, respectively.

The identity between the *H. bleekeri* IRF7 amino acid sequence and those in other fish species ranged from 55.90 to 88.94%, being highest for *Oncorhynchus mykiss*. Its similarity with mammalian IRF7 was low, at 25.8% for humans and 30.4% for mice.

The identity between *H. bleekeri* IRF9 and that in other fish species was at 60.32–97.17%, with *Salvelinus fontinalis* exhibiting the highest identity. IRF9 exhibited high identity with IRF9 in *Salmo* and *Oncorhynchus*.

In addition, we analyzed the identity of DBD region between *H. bleekeri* and other species. The identity of IRF DBD region between *H. bleekeri* and other species is higher than the identity of entire IRF region, especially between species that are more distantly related.

### 3.5. Phylogenetic Analysis

**Figure 4:**
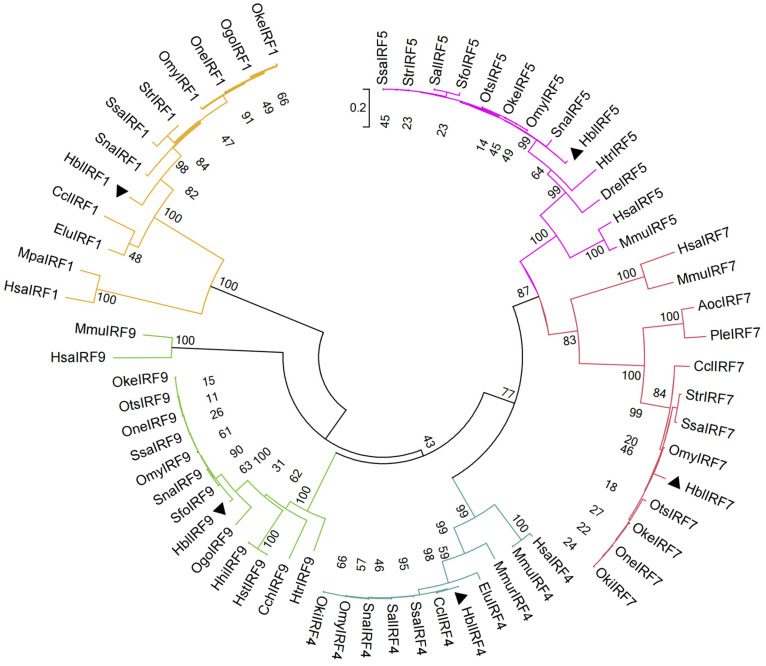
Phylogenetic tree of IRFs based on amino acid sequences. Triangles indicate IRFs of *H. bleekeri*. Hbl: *H. bleekeri*, Ssa: *S. salar*, One: *Oncorhynchus nerka*, Omy: *O. mykiss*, Ots: *Oncorhynchus tshawytscha*, Sfo: *S. fontinalis*, Sna: *S. namaycush*, Oke: *O. keta*, Ogo: *Oncorhynchus gorbuscha*, Htr: *Hypomesus transpacificus*, Cch: *Chanos chanos*, Hst: *Hippoglossus stenolepis*, Hhi: *Hippoglossus hippoglossus*, Hsa: *Homo sapiens*, Mmu: *Mus musculus*, Elu: *Esox lucius*, Ccl: *C. clupeaformis*, Mpa: *Mus pahari*, Sal: *Salvelinus alpinus*, Oki: *Oncorhynchus kisutch*, Mmur: *Myripristis murdjan*, Dre: *Danio rerio*, Aoc: *Amphiprion ocellaris*, Ple: *Plectropomus leopardus*.

The NJ phylogenetic tree was examined by selecting the representative IRF sequences of vertebrates at different evolutionary positions (Figure 4). This tree separates the *H. bleekeri* IRFs into the IRF1, IRF3, IRF4 and IRF5 subfamilies. In *H. bleekeri*, IRF4 and IRF9, on two branches within the IRF4 subfamily, are more closely related to each other than to other IRF members in the IRF phylogeny, implying that IRF4 and IRF9 evolved from a common ancestral gene, separating later from the rest of IRF family and each diverging into small branches. In *H. bleekeri*, IRFl is closely related to, and clustered with, *S. salar*, *S. namaycush* and *Salmo trutta* IRF1. IRF4 of *H. bleekeri* is closely related to that of *Coregonus clupeaformis* and *S. salar*, with which it is clustered onto one branch, and its IRF5 is closely related to that of *S. namaycush* and *O. mykiss*. The IRF7 of *H. bleekeri* is closely clustered with that of *O. mykiss*, while its IRF9 is clustered with those of *S. namaycush* and *S. fontinalis*. In each IRF subfamily, the member in *H. bleekeri* is clustered more with those of other salmonids than with homologous members in other fish species, whereas the mammalian IRF member is located on a separate branch.

### 3.6. IRF Gene Expression in Healthy Tissue

**Figure 5:**
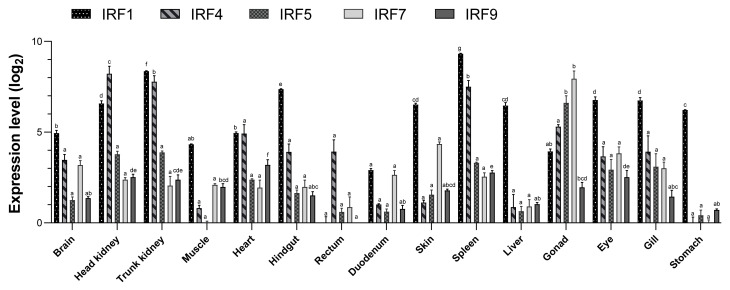
Analysis of *IRF1*, *IRF4*, *IRF5*, *IRF7* and *IRF9* mRNA abundance in 15 tissues as measured by real-time PCR, n = 3. The gene expression level is homogenized by base-2 logarithm. Different lowercase letters represent a significant difference.

Based on real-time PCR, the IRF genes were widely expressed in the skin, brain, gills, head kidney, trunk kidney, liver, spleen, eye, gonad, heart, stomach, duodenum, rectum, hindgut and muscles (Figure 5). *IRFl* was highly expressed in the spleen and kidney, with the highest expression in the spleen, followed by the trunk kidney, hindgut and head kidney. *IRF1* was also highly expressed in the gills, skin, liver and eyes, with the lowest expression in the rectum. *IRF4* expression was highest in the head kidney, followed by the trunk kidney and spleen, but relatively low in other tissues. *IRF5* and *IRF7* expression was highest in the gonads, high in the head kidney, trunk kidney, gill and spleen, but relatively low in other tissues. *IRF9* expression was higher in the heart, spleen, head kidney, trunk kidney and eyes, and lowest in the rectum. The IRF genes were all highly expressed in the immune-related tissues, i.e., the spleen, head kidney and trunk kidney.

### 3.7. Changes in IRF Gene Expression Following LPS Induction

**Figure 6:**
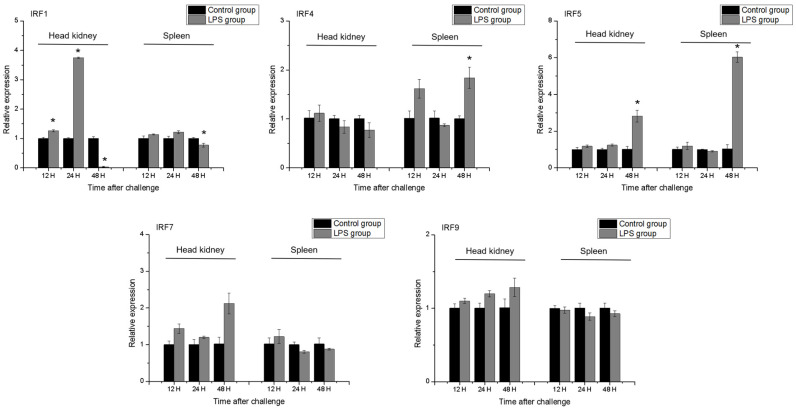
Relative expression of *IRF1*, *IRF4*, *IRF5*, *IRF7* and *IRF9* in head kidney and spleen primary cells after stimulation with LPS. Statistical significance is denoted with “*” (*p* < 0.05), n = 3. The gene expression is homogenized by log_2_ (expression + 1).

LPS stimulation altered IRF gene expression in the head kidney and spleen, to different extents (Figure 6). In the head kidney, *IRF1* was significantly upregulated 12 and 24 h after LPS induction, but its expression had declined by 48 h post-challenge. Expression in the spleen was significantly downregulated 48 h after induction. In the head kidney, LPS induction did not significantly alter *IRF4* expression, whereas in the spleen, *IRF4* was significantly upregulated 48 h after LPS induction. In the spleen and head kidney, *IRF5* was significantly upregulated at 48 h after LPS stimulation, but not at the other time-points. *IRF7* and *IRF9* expression in the spleen and head kidney was not significantly altered by LPS induction.

### 3.8. Changes in IRF Gene Expression Following Poly(I:C) Induction

**Figure 7:**
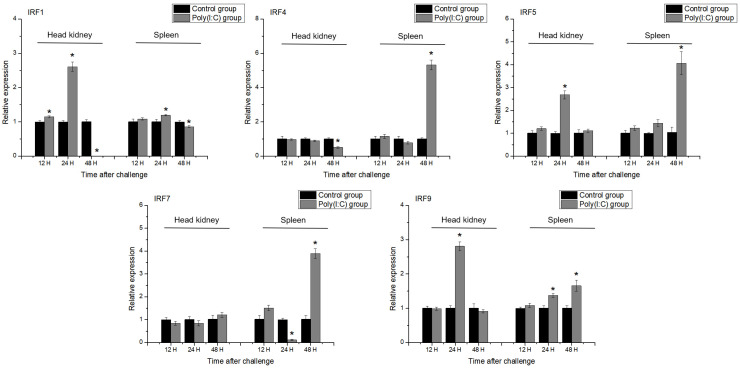
Relative expression of *IRF1*, *IRF4*, *IRF5*, *IRF7* and *IRF9* in head kidney and spleen primary cells after stimulation with poly(I:C). Statistical significance is denoted with “*” (*p* < 0.05), n = 3. The gene expression is homogenized by log_2_ (expression + 1).

Compared to LPS, poly(I:C) induced more significant changes in IRF gene expression in *H. bleekeri* (Figure 7). *IRF1* expression exhibited a similar response after poly(I:C) induction as after LPS induction. In the head kidney, *IRF1* expression was significantly upregulated at 12 and 24 h after induction, but had declined by 48 h. *IRF1* expression in the spleen was significantly upregulated 24 h after induction and significantly downregulated at 48 h. At 48 h after poly(I:C) induction, *IRF4* expression in both the spleen and head kidney was significantly different from that in the control. Following poly(I:C) induction, *IRF4* was downregulated in the head kidney, whereas in the spleen it was upregulated >40-fold; *IRF5* expression was significantly upregulated in the spleen and head kidney after 48 and 24 h, respectively, but not at the other time-points. Poly(I:C) induction did not significantly alter *IRF7* expression in the head kidney, whereas its expression in the spleen was significantly upregulated at 48 h post-challenge. Following poly(I:C) induction, *IRF9* expression was significantly upregulated in the head kidney at 24 h and in the spleen at 24 and 48 h.

## 4. Discussion

IRFs, transcriptional regulatory factors that modulate IFN and ISG expression, play important roles in vertebrate immune defenses. In this study, we cloned five IRF members in *H. bleekeri* and analyzed their sequences and expression. A phylogenetic tree based on the predicted IRF amino acid residues was constructed for fish and other vertebrates, and the evolutionary positions of IRF1, IRF4, IRF5, IRF7 and IRF9 were determined. Tissue-specific expression of *IRF1*, *IRF4*, *IRF5*, *IRF7* and *IRF9* was examined. The effects of LPS-and poly(I:C) induction on *IRF* expression were analyzed. These findings lay the foundation for elucidating the role of these IRF genes in antiviral infection.

Based on these findings, these IRF members exhibited the conserved structure of fish IRFs in *H. bleekeri*. Sequence comparison revealed that *H. bleekeri* IRFs were highly homologous to those of other bony fish (particularly other Salmonidae), to which they are closely related, whereas they exhibited relatively low homology with the IRFs of higher mammals. The *H. bleekeri* IRFs exhibit two domains that are conserved in other vertebrates, namely, the DBD at the N-terminus and IAD at the C-terminus. The IRF5 C-terminus contains a VAD region, whereas that of IRF7 contains a serine-rich region, similar to that observed in Japanese flounder [39]. All IRFs contain an N-terminal DBD with four to five tryptophan repeat motifs [40]. This DBD region forms a helix-turn-helix region that binds to the 5′-GAAA-3′ sequence in the promoter, thereby regulating type I IFN transcription and expression [41]. Three of these tryptophan residues are critical for the interactions between DNA, proteins and IRF molecules, affecting orientation and stabilization during binding to the ISRE/IRF–EGAAA core sequence [11,42]. Here, the DBDs of IRF1, IRF4, IRF5, IRF7 and IRF9 contain 6, 5, 5, 4 and 5 tryptophan repeats, respectively. IRF7 exhibits only four tryptophan residues in the DBD, with the second tryptophan residue replaced by other amino acids, as in other fish [38]. Whether the absence of the second tryptophan residue affects DNA-binding specificity requires further investigation.

In addition to exhibiting conserved DBDs, the IRFs also exhibit conserved IADs. The IAD, first discovered in IRF8, interferes with the activation of IRF7 [43]. The IAD has previously been identified in all IRFs other than IRF1 and IRF2, and its sequence is highly conserved [44]. Here, however, we identified an IAD in all five IRFs in *H. bleekeri*. The SRD, a highly conserved region at the IRF7 C-terminus, plays an important role in virus-induced phosphorylation and is responsible for the interaction between IRF3 and IRF7 [45].

To determine the evolutionary relationships among IRFs, we conducted a phylogenetic analysis including IRF1, 4, 5, 7 and 9 sequences for various other vertebrates. This analysis separates the IRF family into four subfamilies, namely, IRF1, IRF3, IRF4 and IRF5. IRF4 and IRF9 are located in the IRF4 family, whereas IRF7 belongs to the IRF3 subfamily. IRF4 and IRF9 evolved from a common ancestor gene and subsequently diverged from the other IRF members. IRF4 and IRF9 are the most closely related of these IRFs. Finally, the IRF genes in *H. bleekeri* are closely homologous to those of other salmonids, which is consistent with their evolutionary status.

*IRF* was expressed in all of the *H. bleekeri* tissue types examined, indicating that these five genes are constitutively expressed in the 15 tissue types examined. This is consistent with the *IRF* expression profiles of fish such as Atlantic cod [24], large yellow croaker [46], Red Sea Bream (*Pagrus major*) [17], Pacific cod [22], half-smooth tongue sole [23], mandarin fish [14], barbel chub [47] and blunt snout bream [48], in which all *IRFs* were broadly expressed in all of the tested tissue types. *IRF1* and *IRF4* exhibited high expression in immune-related tissues such as the head kidney, trunk kidney and spleen. The spleen and head kidney are important immune organs in fish, suggesting that *IRF1* and *IRF4* are involved in immune responses in *H. bleekeri*. Similar results have been observed in other fish species, with high expression of *IRF1* and *IRF4* in the spleen and head kidney of Atlantic salmon [49], half-smooth tongue sole [23] and large yellow croaker [16]; in the spleen and gut of mandarin fish [14]; and in the head kidney of golden pompano [26]. Here, *IRF1* was also highly expressed in the hindgut, an important mucosal immune-related tissue containing many lymphocytes, indicating that *IRF1* is highly expressed in lymphocyte-rich tissues, and especially in the head kidney, spleen and posterior intestine. In mammals, *IRF1* is strongly inducible by IFN-γ and binds promoter sequences known as IFN-stimulated responsive element (ISRE) and is essential for CIITA induction by IFN-γ [50]. CIITA is a non-DNA-binding transcriptional coactivator essential for both constitutive and IFN-γ-inducible MHC II expression [51,52]. Interestingly, our previous study showed very similar expression patterns of *MHC II* and *IRF1*, with the dominant expression of *MHC II* found in spleen, kidney and hindgut of *H. bleekeri* [53]. In zebrafish, the expression of *MHC II* is regulated by *IFN* and *IRF1* [54]. The simultaneous high expression of *MHC II* and *IRF1* in tissues with dense immune cells suggests that *IRF1* may also have a regulatory effect on *MHC II* expression in *H. bleekeri*.

Surprisingly, both *IRF5* and *IRF7* were highly expressed in the gonads, while *IRF5* was also highly expressed in immune-related tissue. Unlike that of *IRF1* and *IRF4*, tissue-specific patterns of *IRF5* and *IRF7* expression vary substantially among fish. *IRF5* expression was highest in the blood in mandarin fish [14], in the liver and brain in red sea bream [17], in the gills in common carp [18], in muscle in large yellow croaker [16] and in the gonad and muscle in zebrafish [55]. *IRF7* was highly expressed in the liver in mandarin fish [14], spleen and kidney in crucian carp [56] and in the gills and heart in large yellow croaker [16] and barbel chub [47]. Interestingly, *IRF7* was not expressed in all tissues of rainbow trout [57], a species related to *H. bleekeri*; in Atlantic salmon, another related species, *IRF7* expression was highest in the gonads [49], indicating that *IRF7* may have similar functions in *H. bleekeri* and Atlantic salmon. High expression of *IRF* genes has also been observed in the gonads of other species [58], indicating that *IRF* might participate in reproductive process beyond immune function. Here, we observed high *IRF9* expression in immune-related tissue and in the heart. Similarly, *IRF9* was highly expressed in the heart in large yellow croaker [16] and in the spleen in mandarin fish [14]; in Asian sea bass, in contrast, its highest expression was in the muscle [20], suggesting that *IRF9* may have different functions in different species of fish. In mammals, IRFs are susceptible sensors of cardiometabolic stress and function as powerful mediators of related diseases [59,60]. Recent clinical and experimental studies have identified critically important roles of the IRFs in cardiovascular diseases, arising from their participation in divergent and overlapping molecular programs beyond the immune response [61]. Among IRFs, IRF9-deficient mice exhibit especially severely impaired production of IFN-α and IFN-β induced by viral infection and enhanced susceptibility to infection with encephalomyocarditis virus [62]. Therefore, high expression of *IRF9* in the heart of *H. bleekeri* might suggest it plays roles in maintaining the normal physiological function of the heart in fish and interferon might respond to other pathologies such as cardiac disease besides innate immunity. 

Poly(I:C) and LPS were used as stimulants to detect *IRF* gene expression in the spleen and head kidney at different time points. Here, *IRF1* expression was significantly upregulated by LPS and poly(I:C) treatment at 24 h. Similar results for *IRF1* have been observed in fish such as half-smooth tongue sole [23], Atlantic salmon [49] and mandarin fish [14]. Here, *IRF1* showed early response to stimulants in head kidney. Similarly, we found the significant upregulation of *MHC II β* in head kidney at early stage after LPS stimulation in *H. bleekeri* [53]. In mammals, LPS was reported to induce endogenous IFN-γ production, which greatly increased MHC expression in the kidneys of normal mice, in contrast to *IRF1* knockout mice [63]. The result further indicated *IRF1* might play a role in the regulation of the expression of *MHC II* in *H. bleekeri*. Paradoxically, *IRF1* expression was significantly downregulated in both the head kidney and spleen after treatment with LPS and poly(I:C) at 48 h after induction. Similarly, in large yellow croaker, *IRF* expression in the liver was significantly upregulated soon after LPS and poly(I:C) treatment, but was significantly downregulated at 48 h [46]. In Japanese flounder, *IRF8* expression first increased and then declined significantly in the spleen following stimulation [64]. In large yellow croaker, *IRF1* was significantly downregulated in the head kidney after poly(I:C) treatment [65]. This expression pattern may be caused by a random stress response in the control group, as observed in Pacific cod [22], or at this time, poly(I:C) and LPS stimulation may disrupt immune function and alter the transcription of related genes. 

*IRF4* and *IRF5* expression was upregulated in response to poly(I:C) and LPS induction, and, unlike *IRF1*, was significantly more reactive in the spleen than in the head kidney. In Atlantic cod, poly(I:C) treatment significantly upregulated *IRF4* in the spleen [24], whereas in mandarin fish, both poly(I:C) and kidney necrosis virus stimulation upregulated *IRF* expression [14]. These results reveal the potential antiviral and antibacterial roles of *IRF4* and *IRF5* in *H. bleekeri*. *IRF7* and *IRF9* exhibited immune responses to poly(I:C) but not LPS stimulation. Poly(I:C) activated *IRF7* expression in TO cells in Atlantic salmon [49] and upregulated *IRF7* in the head kidney of Japanese flounder [39]. *IRF7* was significantly upregulated in the spleen and kidney under nervous necrosis virus (NNV) stimulation [20]. Poly(I:C) treatment upregulated *IRF9* in both the spleen and head kidney of miiuy croaker [21] and in various tissue types in common carp [19]. Nonetheless, few prior studies have examined *IRF* expression following LPS treatment. Our findings indicate that *IRF7* and *IRF9* are important regulatory factors in poly(I:C)-induced immune responses in the IFN system, and confirm the importance of *IRF* genes in immune responses to pathogens, particularly to viruses. These results provide a reference for the study of signal transduction pathways in the IFN system and in immune defense against viral diseases in *H. bleekeri*.

In this study, we found most IRFs (*IRF1*, *IRF5* and *IRF9*) expressed soonest and most strongly in the head kidney at an early time-point in response to poly(I:C) stimulation, revealing the importance of this organ’s important immunity-related function and how easily it responds to challenge. In the later period after stimulation, they were no longer upregulated in head kidney, whereas the expression levels of *IRF4*, *IRF5*, *IRF7* and *IRF9* were significantly upregulated in spleen at 48 h after stimulation. In mammals, IRF7 plays a major role in the late phase of the induction of IFNs [66]. In fish, the head kidney is a primary lymphoid organ and the spleen is characterized a secondary immune organ. The expression pattern of IRFs in this study might indicate that the IFN pathway in the head kidney is activated first after virus stimulation, and the spleen plays a role in the later stage. IRF signaling in the immune system largely overlaps with the pattern recognition receptor (PRR) and IFN pathways. Different IRF members respond to different PRRs, primarily TLRs, depending on the type and location of the invading pathogen and the upstream cascades [67]. Our results indicate that IRF might bind to different receptors in different infected tissues and play roles at different times to induce the transcription of IFNs and the production of other receptors to coordination of the elimination of pathogens.

## 5. Conclusions

In conclusion, we characterized five IRFs from *H. bleekeri*, each exhibiting a typical DBD and IAD. The amino acid sequences of these *H. bleekeri* IRFs exhibited high homology with those of other salmonids. Based on phylogenetic analysis, these IRFs occur within four subfamilies, with IRF4 and IRF9 belonging to two branches of the IRF4 subfamily. Based on their tissue-specific distribution, these *IRF* genes were widely expressed in all tested tissues, with high expression in immune-related tissues. Poly(I:C) induction significantly altered the expression of all of these *IRF* genes in *H. bleekeri*, whereas LPS challenge induced only *IRF1*, *IRF4* and *IRF5*. Based on these findings, IRFs may participate in antiviral responses in *H. bleekeri*. These findings provide reference information for studying the role of the IFN system in the prevention and treatment of viral diseases in fish.

## Figures and Tables

**Table 1:** Primers designed for full-length cloning and real-time PCR.

Gene Name	Primer Name	Sequence (5′-3′)	Annealing Temperature	Application	Size of PCR Product (bp)
IRF1	IRF1-F	GCAGCTAGCTGAACCATGC	52	Cloning	984
	IRF1-R	GGTTCTGTACAGGGTCTGAG			
IRF4	IRF4-F	GGGTTTCGAGTTTCAGCGAG	52	Cloning	1472
	IRF4-R	TCAGTCCTGCAGGGTGTTG			
IRF5	IRF5-F	TTCTCCTCCCTGCGTGTTG	52	Cloning	1609
	IRF5-R	TCACGGTCCGTTTGTGGC			
IRF7	IRF7-F	GACGTGTCTCAACATTCAAGATGC	52	Cloning	1203
	IRF7-R	TCTAGAAGTACTGCCCCATGG			
IRF9	IRF9-F	ATGGCATCTGGGAGAATTCGCT	52	Cloning	1363
	IRF9-R	AGACTCGGGACATAGCCAGT			
IRF1	IRF1-qF	CATCAGTGGATTGGTGTGGGTGG	58	Real-time PCR	162
	IRF1-qR	GGGTCTGGTTTAGTCTCGCCTTG			
IRF4	IRF4-qF	GCGAGGCTGGATGAAGGAC	58	Real-time PCR	167
	IRF4-qR	GGGCGTCAGTAGGGCTGTA			
IRF5	IRF5-qF	AAGATGGGCTCCCTGACGGTGA	58	Real-time PCR	155
	IRF5-qR	CGCTGCTTCTCGTTCTGGATGTCG			
IRF7	IRF7-qF	CTGCTCAACCTGCCTGCTG	58	Real-time PCR	84
	IRF7-qR	GGTCTTTCGCATCTCGCTCC			
IRF9	IRF9-qF	GCCACATTCAACCACGACG	58	Real-time PCR	157
	IRF9-qR	CTCCTCTCCGAAACACAAGGT			
EF1α	EF1α-qF	TGGAGACAGCAAGAACGACC	62.6	Real-time PCR	126
	EF1α-qR	ATGTGAGCGGTGTGGCAAT			

## Data Availability

The data presented in this study are available on request from the corresponding author.

## Supplementary Materials

The following supporting information can be downloaded at https://www.mdpi.com/article/10.3390/genes15111418/s1, Figure S1: Multiple alignment of IRF1, IRF4, IRF5, IRF7 and IRF9 from *H. bleekeri* with other vertebrate; Table S1: General structures of IRFs in *H. bleekeri*; Table S2: Analysis of amino acid identity of IRFs between *H. bleekeri* and other species.
