# Supplementary material for: Interferon Regulatory Factors (IRF1, IRF4, IRF5, IRF7 and IRF9) in Sichuan taimen (Hucho bleekeri): Identification and Functional Characterization
Source: Genes (Basel). 2024 Oct 31;15(11):1418. doi: 10.3390/genes15111418 (PMC11593489; doi:10.3390/genes15111418)
Supplement: Supplementary file 1 [file genes-15-01418-s001.zip › genes-3257690-supplementary/Figure S1.docx]

IRF1

DBD

IAD

IRF4

DBD

IAD

IRF5

DBD

VAD

IAD

DBD

IRF7

SRD

IAD

IRF9

DBD

IAD

**Figure. S1. Multiple alignment of IRF1, IRF4, IRF5, IRF7 and IRF9 from *Hucho bleekeri* with other vertebrate.** Hbl: *Hucho bleekeri*, Ssa: *Salmo salar*, One: *Oncorhynchus nerka*, Omy: *Oncorhynchus mykiss*, Ots: *Oncorhynchus tshawytscha*, Sfo: *Salvelinus fontinalis*, Sna: *Salvelinus namaycush*, Oke: *Oncorhynchus keta*, Ogo: *Oncorhynchus gorbuscha*, Htr: *Hypomesus transpacificus*, Cch: *Chanos chanos*, Hst: *Hippoglossus stenolepis*, Hhi: *Hippoglossus hippoglossus*, Hsa: *Homo sapiens*, Mmu: *Mus musculus*, Elu: *Esox lucius*, Ccl: *Coregonus clupeaformis*, Mpa: *Mus pahari*, Sal: *Salvelinus alpinus*, Oki: *Oncorhynchus kisutch*, Mmur: *Myripristis murdjan*, Dre: *Danio rerio*, Aoc: *Amphiprion ocellaris*, Ple: *Plectropomus leopardus*.
