# Supplementary material for: Interferon Regulatory Factors (IRF1, IRF4, IRF5, IRF7 and IRF9) in Sichuan taimen (Hucho bleekeri): Identification and Functional Characterization
Source: Genes (Basel). 2024 Oct 31;15(11):1418. doi: 10.3390/genes15111418 (PMC11593489; doi:10.3390/genes15111418)
Supplement: Supplementary file 1 [file genes-15-01418-s001.zip › genes-3257690-supplementary/Table S1.doc]

*Table S1. General structures of IRFs in Hucho bleekeri*

| Gene | cDNA length (bp) | ORF length (bp) | Molecular weight (KDa) | Isoelectric point | Tryptophan repeat | Binding sites |
| --- | --- | --- | --- | --- | --- | --- |
| IRF1 | 1605 | 939 | 35.6 | 5.13 | Trp11, Trp26, Trp38, Trp46, Trp58, Trp77 | Arg82, Cys83, Asn86, Ser87 |
| IRF4 | 2156 | 1407 | 53.4 | 5.7 | Trp22, Trp37, Trp49, Trp69, Trp88 | Arg82, Cys83, Asn86, Lys87 |
| IRF5 | 2494 | 1581 | 59.5 | 5.34 | Trp14, Trp29, Trp41, Trp61, Trp80 | Arg82, Cys83, Asn86, Lys87 |
| IRF7 | 1866 | 1182 | 45.4 | 5.98 | Trp11, Trp36, Trp55, Trp72 | Arg82, Ser83, Asn86, Ser87 |
| IRF9 | 1528 | 1323 | 49.3 | 6.33 | Trp15, Trp30, Trp42, Trp62, Trp80 | Arg82, Cys83, Asn86, Lys87 |
