# Supplementary material for: Interferon Regulatory Factors (IRF1, IRF4, IRF5, IRF7 and IRF9) in Sichuan taimen (Hucho bleekeri): Identification and Functional Characterization
Source: Genes (Basel). 2024 Oct 31;15(11):1418. doi: 10.3390/genes15111418 (PMC11593489; doi:10.3390/genes15111418)
Supplement: Supplementary file 1 [file genes-15-01418-s001.zip › genes-3257690-supplementary/Table S2.doc]

**Table S2.** Analysis of amino acid identity of IRFs between *H. bleekeri* and other species.

| **Gene** | **Species** | **NCBI accession No.** | **Identity of entire sequence** | **Identity of DBD domain** |
| --- | --- | --- | --- | --- |
| IRF1 | *Oncorhynchus keta* | XP_052313595.1 | 87.50% | 96.49% |
|  | *Oncorhynchus gorbuscha* | XP_046162825.1 | 86.86% | 96.49% |
|  | *Oncorhynchus nerka* | XP_029482952.1 | 86.22% | 96.49% |
|  | *Oncorhynchus mykiss* | XP_021478073.2 | 86.22% | 95.61% |
|  | *Salmo salar* | NP_001117117.1 | 86.54% | 93.91% |
|  | *Salvelinus namaycush* | XP_038844947.1 | 86.54% | 95.65% |
|  | *Salmo trutta* | XP_029546388.1 | 85.90% | 93.91% |
|  | *Esox lucius* | XP_010899164.2 | 69.33% | 89.47% |
|  | *Coregonus clupeaformis* | XP_041749567.1 | 67.80% | 92.11% |
|  | *Homo sapiens* | AAA36043.1 | 34.30% | 69.60% |
|  | *Mus pahari* | XP_029402427.1 | 34.60% | 69.60% |
| IRF4 | *Salmo salar* | NP_001133454.1 | 97.44% | 98.40% |
|  | *Salvelinus namaycush* | XP_038854706.1 | 97.86% | 98.40% |
|  | *Salmo trutta* | XP_029606471.1 | 97.22% | 98.40% |
|  | *Salvelinus alpinus* | XP_023853935.1 | 97.65% | 98.40% |
|  | *Oncorhynchus mykiss* | XP_021455274.1 | 97.44% | 98.80% |
|  | *Coregonus clupeaformis* | XP_041705324.1 | 96.79% | 97.60% |
|  | *Oncorhynchus tshawytscha* | XP_024275843.1 | 97.01% | 96.80% |
|  | *Oncorhynchus kisutch* | XP_020348479.1 | 96.79% | 96.80% |
|  | *Esox lucius* | XP_012990445.2 | 87.85% | 94.40% |
|  | *Myripristis murdjan* | XP_029906332.1 | 75.91% | 90.40% |
|  | *Homo sapiens* | KAI4016619.1 | 48.80% | 85.40% |
|  | *Mus musculus* | AAI37714.1 | 52.10% | 84.60% |
| IRF5 | *Salmo salar* | NP_001133324.1 | 94.74% | 98.29% |
|  | *Salmo trutta* | XP_029598521.1 | 94.55% | 97.44% |
|  | *Salvelinus alpinus* | XP_023825107.1 | 93.71% | 96.58% |
|  | *Oncorhynchus tshawytscha* | XP_024232654.2 | 93.81% | 99.15% |
|  | *Salvelinus fontinalis* | XP_055761704.1 | 93.33% | 97.44% |
|  | *Oncorhynchus keta* | XP_035604510.1 | 93.62% | 98.29% |
|  | *Salvelinus namaycush* | XP_038852255.1 | 93.26% | 98.29% |
|  | *Oncorhynchus mykiss* | XP_021419118.1 | 93.46% | 99.15% |
|  | *Hypomesus transpacificus* | XP_046879088.1 | 73.55% | 91.50% |
|  | *Danio rerio* | NP_001314746.1 | 67.98% | 85.50% |
|  | *Homo sapiens* | KAI4015749.1 | 46.60% | 65.00% |
|  | *Mus musculus* | EDL13770.1 | 47.10% | 65.00% |
| IRF7 | *Oncorhynchus mykiss* | CAH56623.1 | 88.94% | 92.52% |
|  | *Oncorhynchus nerka* | XP_029490329.1 | 88.49% | 92.52% |
|  | *Oncorhynchus tshawytscha* | XP_042179164.1 | 87.77% | 92.51% |
|  | *Oncorhynchus nerka* | XP_029490328.1 | 87.65% | 92.52% |
|  | *Oncorhynchus keta* | XP_035612051.1 | 88.01% | 91.59% |
|  | *Oncorhynchus kisutch* | XP_020336522.1 | 87.77% | 90.65% |
|  | *Salmo trutta* | XP_029552514.1 | 87.29% | 94.39% |
|  | *Salmo salar* | NP_001130020.1 | 87.26% | 94.39% |
|  | *Salvelinus alpinus* | XP_023826405.1 | 86.81% | 89.72% |
|  | *Coregonus clupeaformis* | XP_041746910.2 | 76.40% | 86.24% |
|  | *Amphiprion ocellaris* | XP_023149291.2 | 56.94% | 56.90% |
|  | *Plectropomus leopardus* | XP_042351403.1 | 55.90% | 67.60% |
|  | *Homo sapiens* | KAI4069211.1 | 25.80% | 37.40% |
|  | *Mus musculus* | KAI4069209.1 | 30.40% | 39.70% |
| IRF9 | *Salmo salar* | NP_001167190.1 | 96.93% | 96.58% |
|  | *Oncorhynchus nerka* | XP_029482193.2 | 96.70% | 97.44% |
|  | *Oncorhynchus mykiss* | XP_021475909.2 | 96.23% | 97.44% |
|  | *Oncorhynchus tshawytscha* | XP_024257821.1 | 96.70% | 97.44% |
|  | *Salvelinus fontinalis* | XP_055737850.1 | 97.17% | 98.29% |
|  | *Salvelinus namaycush* | XP_038828492.1 | 96.93% | 98.29% |
|  | *Oncorhynchus keta* | XP_035615134.1 | 93.22% | 97.44% |
|  | *Oncorhynchus gorbuscha* | XP_046211653.1 | 80.52% | 86.32% |
|  | *Hypomesus transpacificus* | XP_046880062.1 | 64.88% | 84.48% |
|  | *Chanos chanos* | XP_030629793.1 | 61.01% | 85.47% |
|  | *Hippoglossus stenolepis* | XP_034998333.1 | 60.32% | 78.80% |
|  | *Hippoglossus hippoglossus* | XP_034427777.1 | 60.32% | 78.80% |
|  | *Homo sapiens* | KAI4060268.1 | 30.60% | 68.60% |
|  | *Mus musculus* | AAH12968.1 | 31.20% | 69.20% |

Mammalian species are highlighted in red.
